# lncRNA C2dat2 facilitates autophagy and apoptosis via the miR-30d-5p/DDIT4/mTOR axis in cerebral ischemia-reperfusion injury

**DOI:** 10.18632/aging.202824

**Published:** 2021-04-04

**Authors:** Qian Xu, Ma Guohui, Dandan Li, Fanghui Bai, Jintao Fang, Gui Zhang, Yuxin Xing, Jiawei Zhou, Yugang Guo, Yunchao Kan

**Affiliations:** 1 Henan Provincial Engineering Laboratory of Insects Bio-Reactor, Nanyang Normal University, Nanyang 473000, China; 2 Henan Provincial Nanyang Central Hospital, Nanyang 473000, China; 3 School of Chemistry and Pharmaceutical Engineering, Nanyang Normal University, Nanyang 473000, China

**Keywords:** cerebral ischemia-reperfusion injury, lncRNA, autophagy, apoptosis

## Abstract

Cerebral ischemia-reperfusion injury (CIRI) is an important pathophysiological process of ischemic stroke associated with various physiological and pathological processes, including autophagy and apoptosis. In this study, we examined the role and mechanism of long noncoding RNA CAMK2D-associated transcript 2 (C2dat2) in regulating CIRI *in vivo* and *in vitro*. C2dat2 up-regulation facilitated neuronal autophagy and apoptosis induced by CIRI. Mechanistically, C2dat2 acts as a competing endogenous RNA (ceRNA) to negatively regulate miR-30d-5p expression. More specifically, miR-30d-5p targeted the 3′-untranslated region of DNA damage-inducible transcript 4 (DDIT4) and silenced its target mRNA DDIT4. Additionally, C2dat2 binding with heat shock cognate 70/heat shock protein 90 blocked RNA-induced silencing complex assembly to abolish the miR-30d-5p targeting of DDIT4 and inhibited miR-30d-5p to silence its target mRNA DDIT4. Further analysis showed that C2dat2 knockdown conspicuously inhibited the up-regulation of DDIT4 and Beclin-1 levels and LC3B II/I ratio and the down-regulation of P62 and phosphorylated mammalian target of rapamycin (mTOR)/mTOR and phosphorylated-P70S6K/P70S6K ratio in Neuro-2a cells after oxygen-glucose deprivation/reoxygenation. This study first revealed that C2dat2/miR-30d-5p/DDIT4/mTOR forms a novel signaling pathway to facilitate autophagy and apoptosis induced by CIRI, contributing to the better understanding of the mechanisms of CIRI and enriching the ceRNA hypothesis in CIRI.

## INTRODUCTION

Cerebral ischemia-reperfusion injury (CIRI) is an important pathophysiological process of ischemic stroke involving complex cellular biochemical events, such as inflammation, autophagy, and apoptosis [1]. Restoring blood supply by thrombolytic therapy is the conventional treatment strategy to improve stroke outcomes. However, cerebral injury is often exacerbated by reperfusion after cerebral ischemia, resulting in hemiplegia and infarction [2]. Hence, there is an exigency to identify the precise mechanisms and better treatments against CIRI.

Autophagy is a dynamic process of the self-degradation of cytoplasmic contents and organelles [3, 4]. Increasing evidence reported that autophagy could play a double-edged sword with a pro-death or pro-survival potential role in CIRI [5–9]. The severity of cell injury in CIRI is affected by the degree of autophagy. The moderate activation of autophagy can induce the synthesis of a new protein by degrading damaged proteins [10]. Conversely, excessive autophagy contributes to cell lysis and promotes cell death, accompanied by apoptosis and necrosis [11]. Some investigators have reported that autophagy can reduce infarct volume, neuronal death, and neurological dysfunction after CIRI [12–16]. Others have shown that the excessive activation of autophagy contributes to neuronal death and aggravates the degree of brain injury and that suppressing autophagy attenuated neuronal cell death after CIRI [17–19]. Interestingly, based on the constant interplay of autophagy and apoptosis that may be more complex than previously thought, much evidence also demonstrated that autophagy and apoptosis might share common molecular inducers and regulatory mechanisms [20, 21]. Therefore, discussing the possible mechanisms that underpin the role of autophagy and apoptosis may offer a path to protect against CIRI.

Genome-wide sequencing technologies have revealed that CIRI profoundly impacts the expression of various noncoding RNAs (ncRNAs), such as long ncRNAs (lncRNAs; >200 nt), circular RNA, Piwi-interacting RNA, and microRNAs (miRNAs; 20–25 nt) [22]. Increasing evidence has revealed that lncRNAs function as important mediators to regulate gene silencing in the pathogenesis of stroke [23]. A previous study has also revealed that miRNA binds to the 3'-untranslated region (UTR) of the target mRNA, which has miRNA response elements, leading to the down-regulation of the target gene mediated by Argonaute proteins at the heart of RNA-induced silencing complexes (RISCs), and RISCs provide a platform for the translational repression of the target mRNAs by acting as catalytic engines [24, 25]. These miRNAs or small interfering RNAs (siRNAs) need to form effector ribonucleoprotein complexes (RISC) to fulfill their functions. Heat shock protein 90 (HSP90) and heat shock cognate 70 (HSC70) are molecular chaperones that can bind to other RNAs and act as machinery essential for RISC assembly [24, 26, 27]. Prior studies demonstrated that several lncRNAs crosstalk with other RNAs, including miRNA [28–30]. The miR-30 family is reported to play pivotal roles in many diseases, including stroke [31]. DNA damage-inducible transcript 4 (DDIT4; also known as REDD1, RTP801, and DIG2) is a well-recognized cell stress protein rapidly up-regulated in the absence of oxygen [32], and a negative regulator of the mammalian target of rapamycin (mTOR) autophagy signaling pathway after CIRI [33, 34]. This study involves the bioinformatics analysis of Wu et al.’s data [35]. There are two CAMK2D-associated transcript two lncRNAs (C2dat1 and C2dat2) up-regulated after CIRI [36, 37]. Unlike the nucleus-localized lncRNA C2dat1, C2dat2 is mostly localized in the cytoplasm. Using a bioinformatics approach, C2dat2 is shown to share putative binding sites of miR-30d-5p and the 3'-UTR of DDIT4.

Against this background, this study aimed to uncover novel crosstalk among C2dat2, HSC70/HSP90 conjugate, miR-30d-5p, DDIT4, and mTOR and their implications in autophagy and apoptosis in the context of CIRI and provide experimental and theoretical bases for the further development of novel drugs against CIRI.

## RESULTS

### Bioinformatics analysis

**Figure 1:**
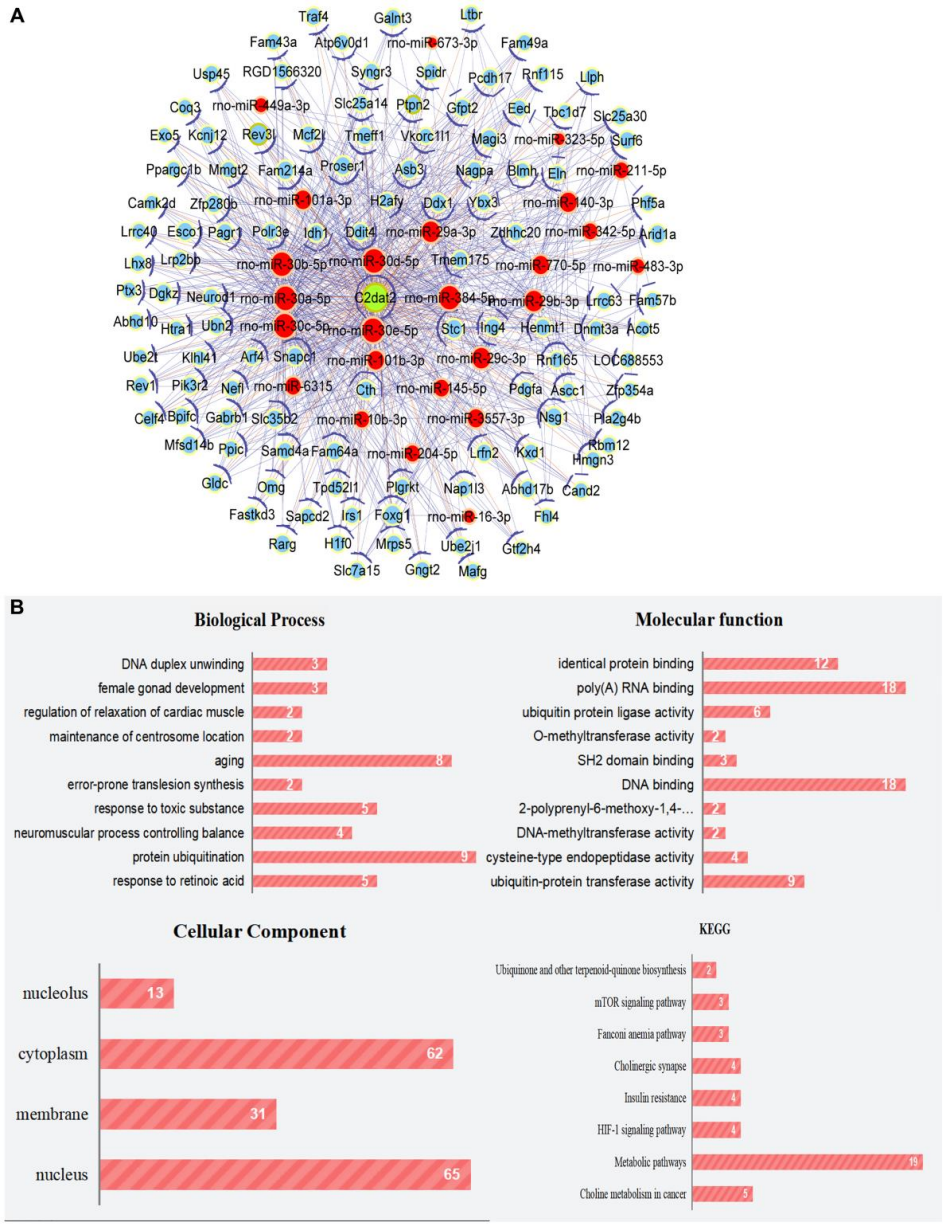
**Cytoscape works with the ceRNA network of C2dat2.** (**A**) ceRNA network of C2dat2. Green nodes denote lncRNAs, blue nodes denote coding genes, and red nodes denote miRNA. The blue lines between two nodes indicate positively correlated interactions between genes, and the orange lines indicate negatively correlated interactions. (**B**) BP, CC, and MF of the GO term and KEGG enrichment analyses of C2dat2-associated mRNA.

Coexpression and competing endogenous RNA (ceRNA) networks may correspond to biological pathways and the functions of miRNAs and mRNA associated with lncRNAs. Thus, C2dat2-associated ceRNA network of the ischemia-reperfusion (I/R) model versus the sham group were constructed. C2dat2-associated ceRNA network were connected to 159 protein-coding genes and 25 miRNAs (Figure 1A; Supplementary Table 1). Significant Gene Ontology (GO) term analysis by Gene Set Enrichment Analysis revealed that mRNAs were expressed in correlation with C2dat2. The pathway enrichment analysis revealed several pathways, including the mTOR and hypoxia-inducible factor-1 signaling pathways, which are involved in cell survival, neuronal autophagy, and apoptosis (Figure 1B). As an exception, the two potential networks between C2dat2 and C2dat1 have also been constructed: the coding-noncoding gene coexpression network (CNC network) and the ceRNA network (Supplementary Figures 1 and 2). As a result, the CNC network revealed many potential lncRNA-mRNA connection pairs, and the ceRNA network revealed many potential lncRNA-miRNA-mRNA connection pairs that might participate in CIRI regulation.

### C2dat2 up-regulation is involved in miR-30d-5p down-regulation in both *in vivo* and *in vitro* models of CIRI

**Figure 2:**
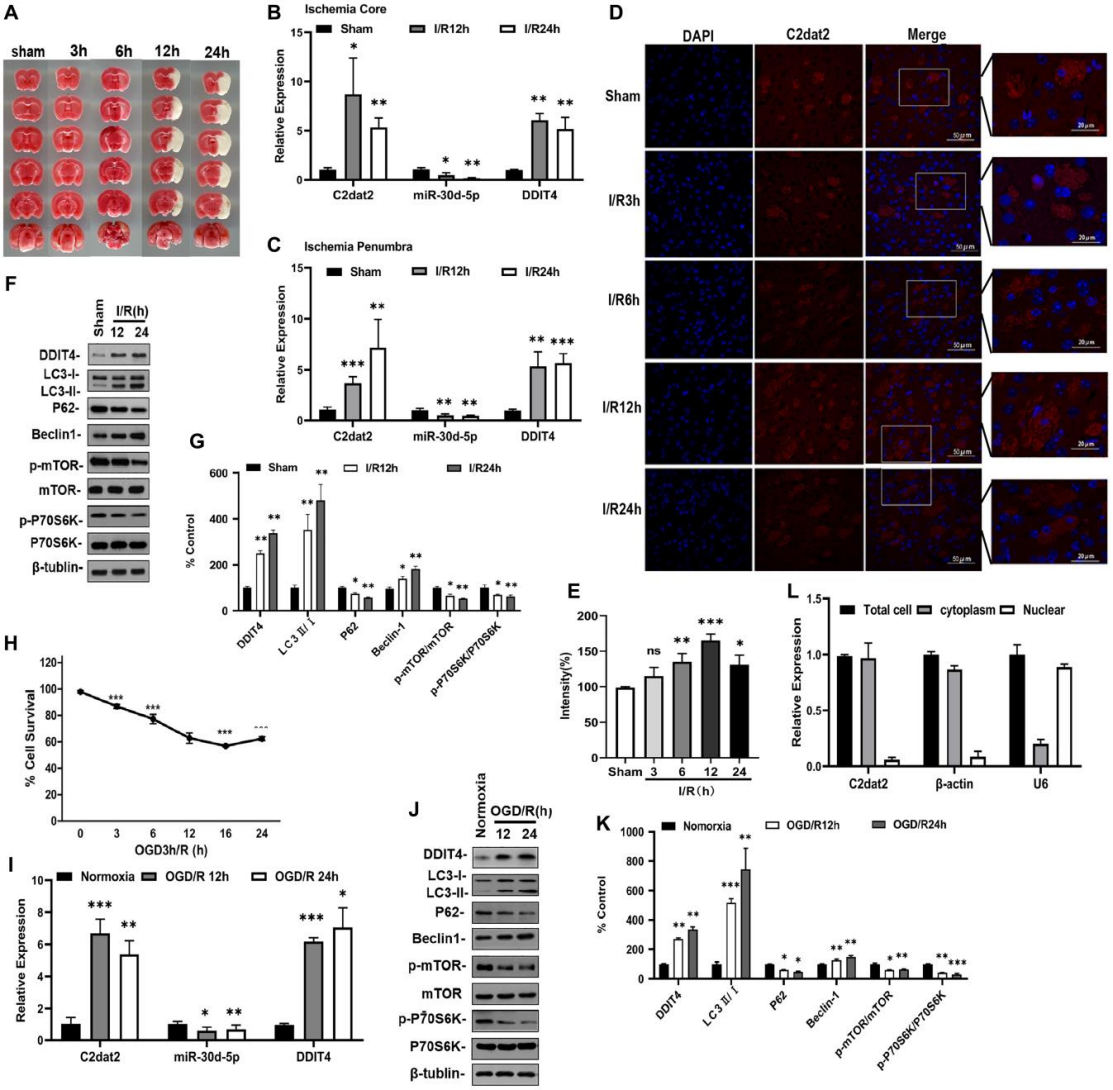
**lncRNA C2dat2, miR-30d-5p, and DDIT4 were involved in the response to I/R-induced injury *in vivo* and *in vitro.*** (**A**) Representative images of TTC-stained brain sections. Representative images in mice after 1 h MCAO and 3, 6, 12, 24, and 36 h reperfusion. (**B**–**C**) C2dat2, miR-30d-5p, and DDIT4 levels in the ischemic core (**B**) and penumbra (**C**) of ischemic and sham tissue were measured via RT-qPCR. GAPDH was used as the control (*n* = 3 per group). (**D**) Representative RNA-FISH images manifesting intracellular localization. (**E**) Intensity of C2dat2 in each group. (**F**) Western blotting showing the expression levels of DDIT4 and autophagy-related protein expression levels (LC3, P62, Beclin-1, p-mTOR, mTOR, p-P70S6K, and P70S6K) in the ischemia penumbra of mice after 1 h MCAO and 12 and 24 h reperfusion. (**G**) Relative protein levels were analyzed. Data are mean ± standard error of the mean (SEM; *n* = 3). (**H**) OGD/R-induced cell death in N2a cells. (**I**) RT-qPCR of C2dat2, DDIT4, and miR-30d-5p levels in OGD-treated cells and normoxia control. (**J**) Typical Western blotting results showed changes in DDIT4 and autophagy-related protein (LC3, P62, Beclin-1, p-mTOR, mTOR, p-P70S6K, and P70S6K) expression levels in N2a cells between normoxia and 12 and 24 h reoxygenation after 3 h OGD. (**K**) Relative protein levels were analyzed. Data are mean ± SEM (*n* = 3). β-Tubulin was blotted as a loading control. ^*^P < 0.05; ^**^P < 0.01; ^***^P < 0.001; ns, not significant versus normoxia or sham. (**L**) Distribution of lncRNA C2dat2 in the cytoplasm and nucleus of N2a cells 24 h after OGD/R. Cell fractionation of U6, β-actin, and C2dat2 in N2a cells. Like β-actin, C2dat2 is expressed in the cytoplasm.

The remaining analysis was performed by inducing mice with middle cerebral artery occlusion (MCAO) for 1 h followed by reperfusion (3, 6, 12, or 24 h; Figure 2A). Brain I/R tissue (core or penumbra) samples were subjected to RNA extraction and reverse transcription-quantitative polymerase chain reaction (RT-qPCR) to measure C2dat2, miR-30d-5p, and DDIT4 expression (Figure 2B and 2C). RT-qPCR analyses showed that the penumbra exhibited a time-dependent up-regulation of C2dat2 (4- to 12-fold; *P* < 0.05) and DDIT4 (4- to 6-fold; *P* < 0.05) and down-regulation of miR-30d-5p (0.8- to 0.5-fold; *P* < 0.05). In the ischemic core, both C2dat2 and DDIT4 transcripts were abruptly up-regulated at 12 h and then gradually down-regulated at 24 h. miR-30d-5p was down-regulated at 12 and 24 h. The fluorescence intensity of C2dat2 in mouse MCAO brain tissue was increased during prolonged I/R by fluorescence *in situ* hybridization (FISH; Figure 2D and 2E). DDIT4 expression was also up-regulated at the protein level compared to the sham group (Figure 2F and 2G).

For *in vitro* ischemia, the Cell Counting Kit-8 (CCK-8) assay was used to detect Neuro-2a (N2a) cell survival. The survival rate was notably decreased in the oxygen-glucose deprivation/reoxygenation (OGD/R) group than in the normoxia group (Figure 2H). Next, C2dat2, DDIT4, and miR-30d-5p expression in the OGD/R group was determined by RT-qPCR. Analogously, as shown in Figure 2I, exposure to OGD/R increased the expression levels of C2dat2 and DDIT4 and reduced that of miR-30d-5p compared to the normoxia group.

Stimulation with OGD/R also triggered a 2.3- to 3.5-fold increase in DDIT4 protein expression relative to the normoxia group (Figure 2J and 2K). To confirm the existence of autophagy in ischemic stroke, the protein levels of Beclin-1, LC3, P62, phosphorylated mTOR (p-mTOR), mTOR, phosphorylated P70S6K (p-P70S6K), and P70S6K were detected via Western blotting. The results indicated that the levels of LC3 II/I ratio and Beclin-1 increased with time, whereas those of P62, p-mTOR/mTOR, and p-P70S6K/P70S6K were reduced in mouse brain after I/R (Figure 2F and 2G) and N2a cells after OGD/R (Figure 2J and 2K), confirming the existence of autophagy in ischemic stroke.

### C2dat2 is predominantly localized in the cytoplasm

The intracellular localization of lncRNA is an important factor in its biological function. RNA-FISH was conducted to estimate the location of C2dat2 under CIRI conditions. This lncRNA was mainly located in the cytoplasm (Figure 2D and 2E). These findings were also confirmed in N2a cells exposed to OGD/R via cell fractionation (Figure 2L). Thus, C2dat2 was shown to be an lncRNA with a cytoplasmic localization. Next, the full-length mouse C2dat2 (cDNA) sequence was acquired via rapid amplification of cDNA ends (RACE) using the mouse I/R12h brain tissue from the surrounding penumbra (Supplementary Figure 3A–3C).

### C2dat2 silencing inhibits OGD-induced neuronal autophagy and apoptosis

**Figure 3:**
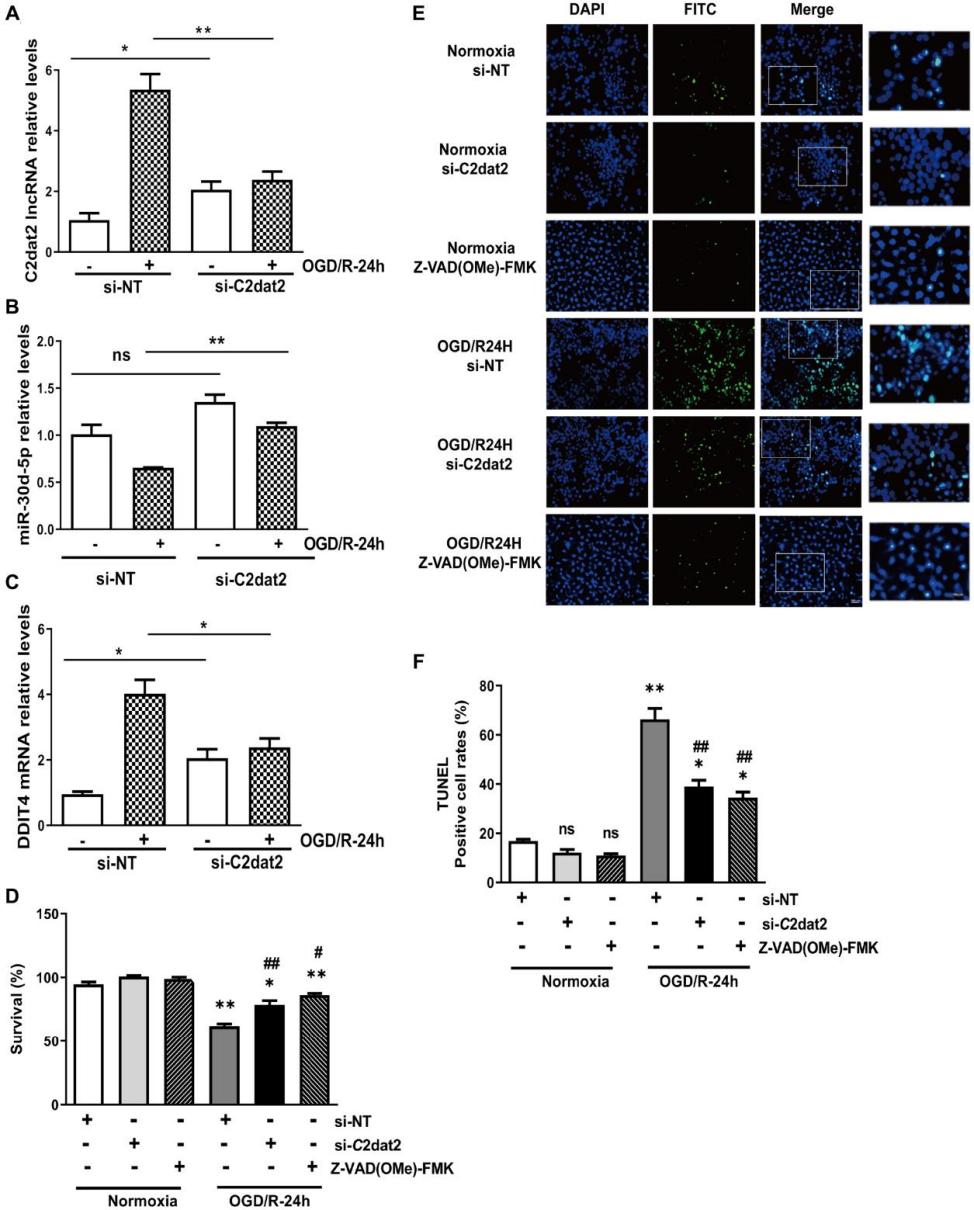
**Effect of C2dat2 down-regulation on apoptosis in N2a cells after OGD/R.** RT-qPCR manifested that C2dat2 knockdown abolished OGD/R-induced (**A**) C2dat2, (**B**) miR-30d-5p, and (**C**) DDIT4 expression in N2a cells. (**D**) Cell survival was examined by the CCK-8 assay after OGD/R with si-NT, si-C2dat2, or Z-VAD(OMe)-FMK (20 μM, 24 h) treatment. (**E**) Representative images of TUNEL staining after OGD/R with si-NT, si-C2dat2, or Z-VAD(OMe)-FMK (20 μM, 24 h) treatment. (**F**) TUNEL-positive cell rate in each group. ^*^P < 0.05; ^**^P < 0.01 vs. normoxia; ^#^*P* < 0.05, ^##^ *P* < 0.01 vs. si-NT; ns, not significant versus normoxia.

Next, a specific smart RNA silencer [including siRNAs and small nucleolar RNAs (snoRNAs)] against C2dat2 was synthesized. Owing to the fact that C2dat2 was mapped to CAMK2d mRNA, the smart silencer was designed by shunning the region that overlapped with CAMK2d (Supplementary Figure 3B). RT-qPCR indicated that C2dat2 knockdown abolished OGD/R-induced C2dat2 (Figure 3A), miR-30d-5p (Figure 3B), and DDIT4 (Figure 3C) in N2a cells. The CCK-8 assay showed that OGD/R-24 h-induced impaired cell viability was significantly alleviated by si-C2dat2 transfection and remarkably alleviated by Z-VAD(OMe)-FMK (20 μM, pre-treatment for 24 h) compared to si-NT (Figure 3D). The results of terminal deoxynucleotidyl transferase-mediated dUTP nick-end labeling (TUNEL) staining were also in agreement with the CCK-8 assay data (Figure 3E and 3F).

**Figure 4:**
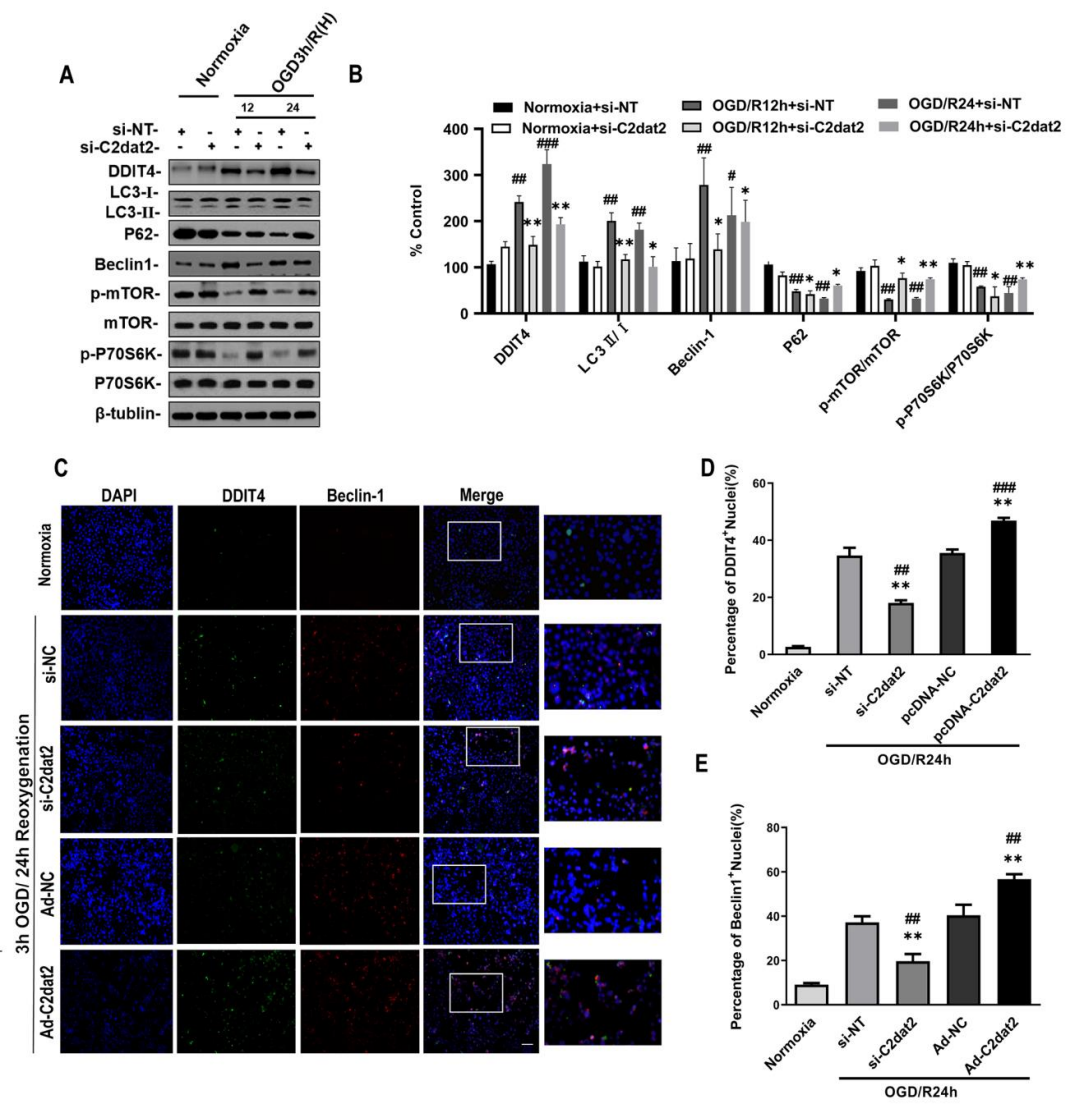
**Effect of C2dat2 down-regulation on autophagy in N2a cells after OGD/R**. (**A**) Relative protein expression levels of DDIT4, LC3, P62, Beclin-1, p-mTOR, mTOR, p-P70S6K, and P70S6K were assessed using Western blotting of N2a cells transfected with si-NC or si-C2dat2 12 and 24 h after OGD/R. (**B**) Relative protein levels were analyzed. Data are mean ± SEM (*n* = 3). β-Tubulin was blotted as a loading control. (**C**) Representative images of double immunofluorescent staining of N2a cells with DDIT4 (green) and Beclin-1 (red). (**D** and **E**) DDIT4 and Beclin-1 expression was examined using immunofluorescence assay. Data are mean ± SEM. ^*^P < 0.05; ^**^P < 0.01 vs. normoxia; ^#^*P* < 0.05; ^##^ *P* < 0.01 vs. si-NT or pcDNA-NC subjected to the same OGD/R (*n* = 3 per group).

Western blotting results indicated that C2dat2 knockdown conspicuously inhibited the increase in OGD/R-induced DDIT4 and Beclin-1 levels and LC3B II/I ratio and the down-regulation of P62 and p-mTOR/mTOR and p-P70S6K/P70S6K ratio (Figure 4A and 4B). Representative images showing the immunofluorescence staining of DDIT4 and Beclin-1 after the groups had been transfected with pcDNA-C2dat2, pcDNA-NC, si-C2dat2, or si-NT followed by OGD/R for 24 h also conformed with the above results, suggesting that C2dat2 knockdown strikingly suppressed autophagy in ischemic stroke (Figure 4C–4E). These outcomes demonstrated that C2dat2 silencing suppresses OGD/R-induced neuronal autophagy and apoptosis.

### C2dat2 overexpression in mouse cerebral cortex can improve MCAO damage of cortical neurons

**Figure 5:**
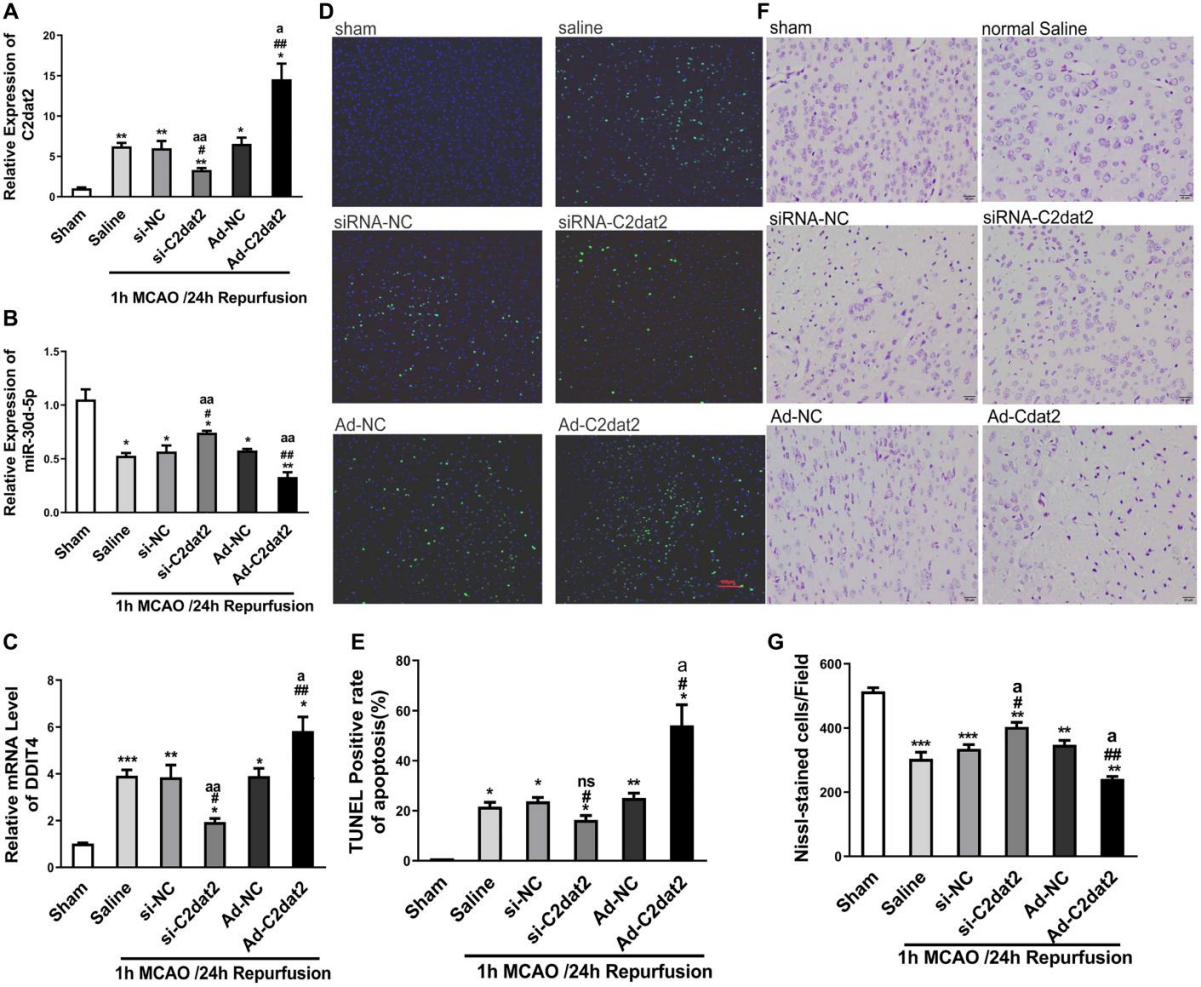
**C2dat2 mediated a catalytic role in ischemic brain injury *in vivo*.** (**A**–**C**) RT-qPCR showed the changes in (**A**) C2dat2, (**B**) miR-30d-5p, and (**C**) DDIT4 expression after infection with si-C2dat2 and Ad-C2dat2 in mice after 1 h MCAO and 24 h reperfusion. (**D**) Apoptosis in the ischemic penumbra was analyzed via TUNEL staining. Representative images showed TUNEL-positive cells in tissue from different groups. (**E**) Apoptotic rate in each group. (**F**) Representative images showing Nissl staining to determine neural cell loss in the ischemic penumbra. (**G**) Quantitative analysis of the effect of C2dat2 on neural cell loss. Data are mean ± SEM. ^*^P < 0.05; ^**^P < 0.01 vs. sham; ^#^*P* < 0.05; ^##^*P* < 0.01 vs. si-NC or Ad-NC; ^a^*P* < 0.05; ^aa^*P* < 0.01 vs. saline (*n* = 3 per group).

To assess whether apoptotic death is involved in the MCAO model, TUNEL assay and Nissl staining were employed. *In vivo*, after 36 h injection, overexpressed C2dat2 (Ad-C2dat2)/control vector [Ad-negative control (NC)]/si-C2dat2/si-NC/saline, mice were induced by I/R. The neurological outcome was tested 24 h after reperfusion. C2dat2, DDIT4, and miR-30d-5p expression was significantly different after knockdown and overexpression (Figure 5A–5C). Moreover, the TUNEL assay strongly demonstrated that I/R-induced neuronal apoptosis could not be enhanced by the injection of Ad-C2dat2 or reduced by si-C2dat2 (Figure 5D and 5E). Nissl staining revealed that Nissl corpuscles in cortical neurons of mice were large in the sham group, highlighting the enhanced function of the synthesized protein of cortical neurons of mice in this group. The loss of Nissl corpuscles in middle neurons was lower after treatment with si-C2dat2, and the loss of Nissl corpuscles in cortical neurons of mice was further aggravated in the Ad-C2dat2 group (Figure 5F and 5G). These findings suggested that C2dat2 overexpression in the mouse cerebral cortex improves the MCAO damage of cortical neurons.

### C2dat2 is a target of miR-30d-5p and negatively regulates its expression

**Figure 6:**
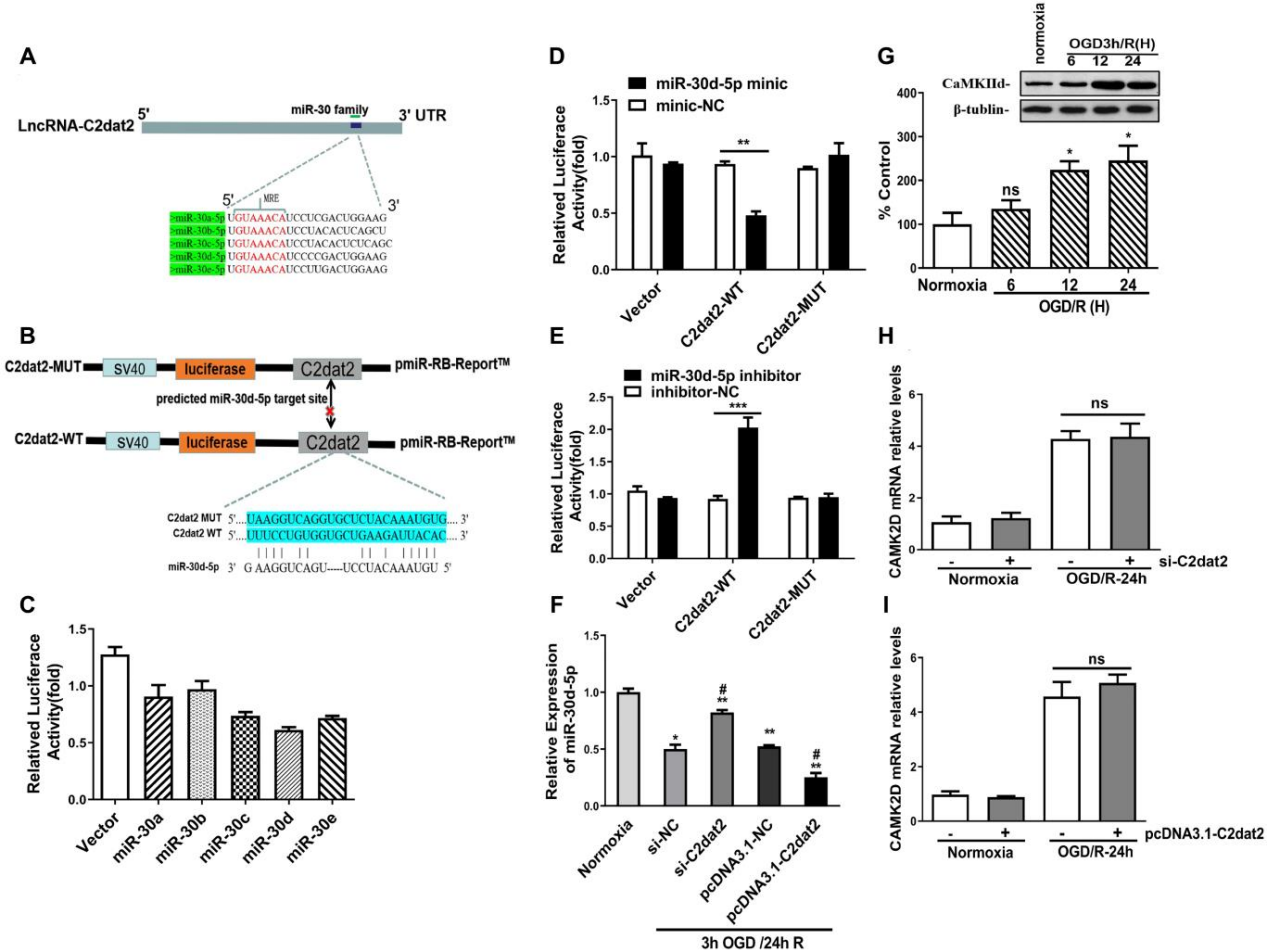
**C2dat2 bound to miR-30d-5p and reduced miR-30d-5p expression.** (**A**) Binding region between the miR-30 family and C2dat2, which was predicted by RNAhybrid software. (**B** and **C**) Dual-luciferase reporter assays demonstrated that C2dat2 directly targeted miR-30d-5p. (**D** and **E**) The luciferase reporter vector carrying C2dat2-WT or the empty vector was cotransfected with the miR-30d-5p mimic or mimic-NC or miR-30d-5p inhibitor or inhibitor-NC into N2a cells as indicated. The relative luciferase activity was detected 48 h after transfection. ^**^P < 0.01 vs. C2dat2-WT. (**F**) The relative expression level of miR-30d-5p was detected via RT-qPCR after C2dat2 knockdown or overexpression. ^*^P < 0.05; ^**^P < 0.01 vs. normoxia; ^##^*P* < 0.05 vs. si-NT or pcDNA-NC, subjected to the same OGD/R (*n* = 3 per group). (**G**) CaMK2d protein expression was measured in N2a cells after OGD/R. (**H** and **I**) The relative expression level of CaMK2d was detected via RT-qPCR after C2dat2 knockdown or overexpression at the normal and OGD/R states.

It is well established that lncRNAs competitively inhibit miRNA expression and functioning in cerebral ischemic insults. To inspect whether C2dat2 exhibits a similar mechanism, the RNAhybrid software was employed and whether the miR-30 family could bind to C2dat2 was explored (Figure 6A–6C). Interestingly, miR-30d-5p mimic drastically repressed the luciferase activity of pmirGLO-C2dat2-wild-type (WT; Figure 6D), while the miR-30d-5p inhibitor noticeably enhanced it (Figure 6E). However, neither had apparent inhibitory effects on pmirGLO-C2dat2-mutant (MUT). miR-30d-5p expression was also substantially reduced by pcDNA-C2dat2 and significantly improved by si-C2dat2 (Figure 6F). Additionally, CaMKIId protein expression was measured in N2a cells after OGD/R. CaMKIId protein noticeably increased within 24 h of reoxygenation (Figure 6G). However, si-C2dat2 did not affect CaMKIId mRNA expression (Figure 6H). Moreover, C2dat2 overexpression slightly up-regulated CaMKIId mRNA levels at the normal and 24 h OGD/R states. Thus, the probability that C2dat2 regulates CaMKIId was ruled out (Figure 6I).

### C2dat2 positively regulated DDIT4 expression by sponging miR-30d-5p

**Figure 7:**
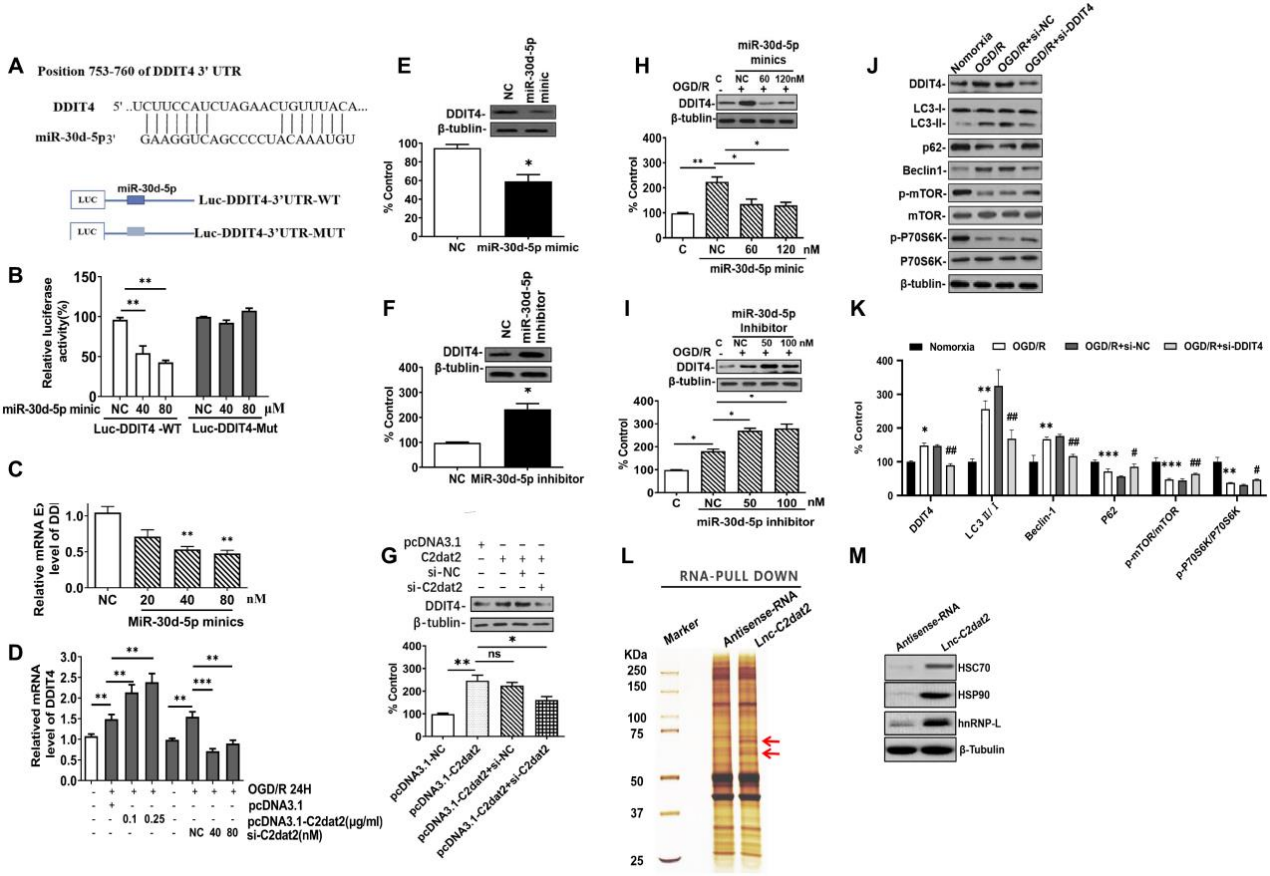
**C2dat2 positively regulated DDIT4 expression by sponging miR-30d-5p.** (**A**) Possible binding sites of miR-30d-5p in DDIT4 3′-UTR. (**B**) luc-DDIT4 3′-UTR-WT or luc-DDIT 3′-UTR-MUT plasmids were cotransfected with NC or miR-30d-5p mimic into HEK 293T cells for 24 h. Luciferase activity was determined. (**C**) After transfection with NC and miR-30d-5p mimic for 48 h, DDIT4 mRNA levels were detected by RT-qPCR. (**D**) N2a cells were treated with OGD/R for 24 h after C2dat2 overexpression or knockdown followed by RT-qPCR of DDIT4 mRNA levels. (**E** and **F**) After transfection with (**E**) miR-30d-5p mimic or (**F**) inhibitor for 48 h, DDIT4 protein levels were assessed via Western blotting. (**G**) After transfection with pcDNA3.1 or C2dat2 (0.1 μg/ml) and then cotransfected with C2dat2 (0.1 μg/ml) and NC or si-C2dat2 (60 nM) for 48 h, DDIT4 protein levels were assessed via Western blotting. (**H** and **I**) After transfection with NC, miR-30d-5p mimic, or inhibitor overnight, N2a cells were treated with OGD/R for 48 h. Western blotting of DDIT4 levels. ^*^P < 0.05; ^**^P < 0.01; ns, not significant vs. normoxia (*n* = 3 per group). (**J**) Relative protein expression levels of DDIT4, LC3, P62, Beclin-1, p-mTOR/mTOR, and p-P70S6K/P70S6K were assessed via Western blotting of N2a cells transfected with si-NT or si-DDIT4 and then treated with OGD/R for 24 h. (**K**) Relative protein levels were analyzed. Data are mean ± SEM. β-Tubulin was blotted as a loading control. (**L**) lncRNA C2dat2 binding with the HSC70/HSPA9 conjugate. RNA pull-down results of lncRNA C2dat2 via silver staining. (**M**) Western blotting confirmed C2dat2, HSC70, and HSP90 expression levels by RNA pull-down of the I/R tissue. ^*^P < 0.05; ns, not significant vs. normoxia; ^##^*P* < 0.05 vs. si-NT (*n* = 3 per group).

In the C2dat2 network, miR-30d-5p interacted with DDIT4 and C2dat2. Therefore, whether miR-30d-5p affected DDIT4 expression was investigated. First, the luciferase reporter assay revealed that DDIT4 3’-UTR-WT inhibited luciferase activity (Figure 7A and 7B). Furthermore, miR-30d-5p mimic down-regulated the mRNA level of DDIT4 (Figure 7C), whereas C2dat2 overexpression abolished this down-regulation (Figure 7D). At the protein level, miR-30d-5p mimic suppressed the protein expression of DDIT4 (Figure 7E), but the miR-30d-5p inhibitor had the opposite effect (Figure 7F). Additionally, pcDNA3.1-C2dat2 up-regulated the protein level of DDIT4, whereas si-C2dat2 abolished this up-regulation (Figure 7G). Under OGD/R conditions, the protein level of DDIT4 was decreased by miR-30d-5p mimic (Figure 7H) but increased by the miR-30d-5p inhibitor (Figure 7I). Therefore, C2dat2 was considered to function as a ceRNA for miR-30d-5p to regulate DDIT4. Next, Western blotting results indicated that DDIT4 negatively regulated mTOR-dependent autophagy and elevated the protein levels of LC3II, Beclin-1, P62, and mTOR pathway proteins, such as p-mTOR and p-P70S6K (Figure 7J and 7K) in DDIT4-silenced N2a cells under OGD/R conditions. The results indicated that C2dat2 functions as a ceRNA for miR-30d-5p to regulate the DDIT4/mTOR signaling pathway.

**Figure 8:**
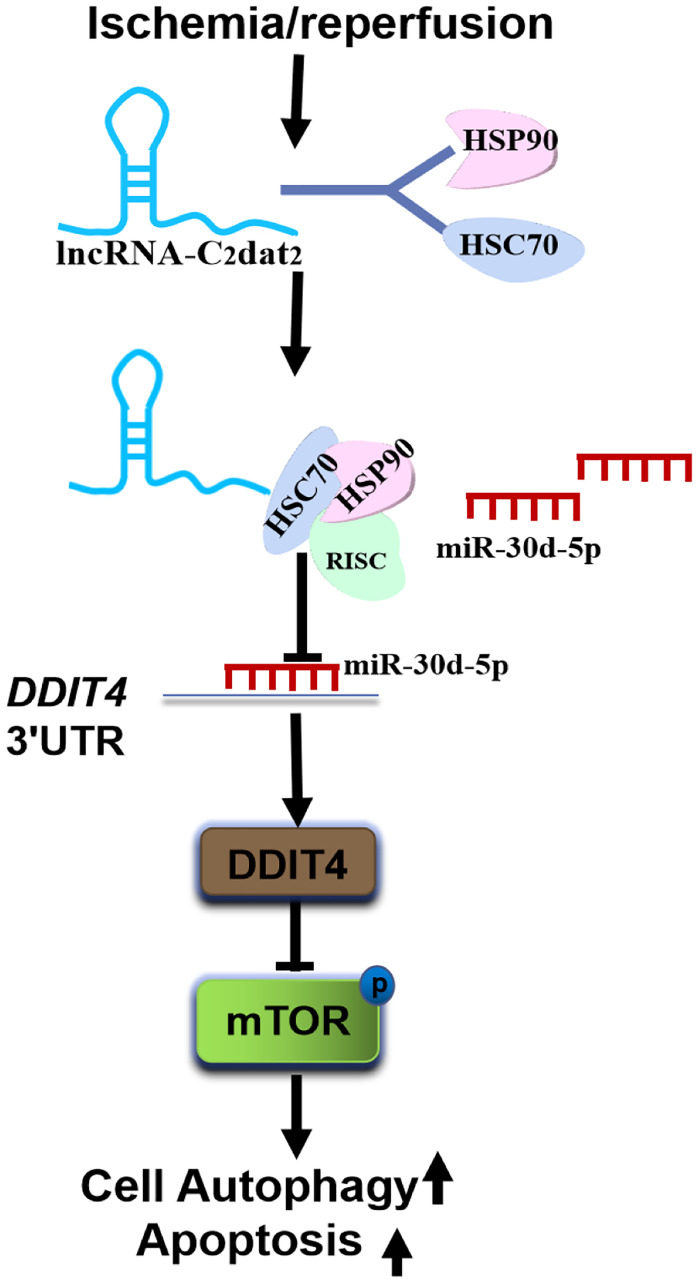
**Diagram depicting the signaling mechanisms of C2dat2 in CIRI**. CIRI induces up-regulated C2dat2 to bind with HSC70/HSP90, blocks the RISC assembly, reduces the chaperone to load miR-30d-5p duplexes into Ago proteins, and inhibits the miR-30d-5p silencing of DDIT4 and then inhibits mTOR signaling pathway protein phosphorylation and facilitates neuronal autophagy and apoptosis.

In addition, the total protein extracts from mouse MCAO penumbra tissue were incubated with biotinylated C2dat2 or the NC and then pulled down with streptavidin. Two distinct bands present in C2dat2 pull-down samples were identified via mass spectrometry (Figure 7L). Moreover, to identify the specific proteins interacting with C2dat2, the mass spectrometry results were analyzed, and the pull-down assay was repeated to test three selected proteins—hnRNPL, HSC70, and HSP90—by Western blotting (Figure 7M). The results suggested that up-regulated lncRNA C2dat2 bound with HSC70 and HSP90. As an a priori hypothesis, HSC70/HSP90 we assumed that may interact between lncRNA C2dat2 and miR-30d-5p. The signaling pathway through which C2dat2 facilitates autophagy and apoptosis via the miR-30d-5p/DDIT4/mTOR axis in response to ischemia is depicted in Figure 8. Taken together, data suggested that the mTOR signaling pathway is a major target of C2dat2-regulated miR-30d-5p/DDIT4 in response to OGD/R in mouse neuronal cells.

## DISCUSSION

Despite advancements in research on ischemic stroke in the last few decades, CIRI remains a major threat to public health worldwide [3]. In recent years, several ncRNAs have been shown to be dysregulated in CIRI [38–43]. Considering their aberrant expression and multiple functions, the roles of lncRNAs functioning as ceRNAs were highlighted as an innovative therapeutic target in CIRI. For instance, Yan et al. found that the lncRNA MEG3 regulates the miR-21/PDCD4 axis in ischemic neuronal death [42]. In addition, Xiao et al. reported that the lncRNA GAS5 modulates Notch1-dependent autophagy through miR-137 in ischemic stroke [38]. It was also uncovered that MALAT1 attenuates neuronal cell death and autophagy through the miR-30a/Beclin-1 axis [39]. Due to the intracellular localization of lncRNA as an important factor in its biological function, Xu et al. described that the nucleus-localized lncRNA C2dat1 could modulate CaMKIId expression to promote neuronal survival through the nuclear factor-κB (NF-κB) signaling pathway in response to I/R [36]. Furthermore, Ye et al. reported that C2dat2 knockdown decreased CaMKIId expression and inhibited the NF-κB signaling activity [42]. In this study, bioinformatics analysis has shown that the CNC network between C2dat1 and C2dat2 confirmed Ye et al.’s results that C2dat1 and C2dat2 might play biological functions through the NF-κB signaling pathway. A ceRNA network between C2dat1 and C2dat2 was also discovered, which showed that the cytoplasmic lncRNA C2dat2 might mainly play biological functions through the ceRNA mechanism in the cytoplasm. More importantly, C2dat2 knockdown markedly ameliorated ischemic injury by suppressing OGD-induced neuronal cell death and MCAO-induced ischemic brain infarction. Besides, miR-30d-5p is a member of the miR-30 (miR-30a/b/c/d/e) family, which is abundantly expressed and plays an important role in ischemic injury [44–48]. This study also determined that miR-30d-5p was decreased in the stroke model both *in vitro* and *in vivo*. C2dat2 knockdown alleviated OGD-induced neuron injury and apoptosis via the up-regulation of miR-30d-5p expression in N2a cells exposed to OGD/R.

As a paramount cell stress protein, DDIT4 has been identified as a regulator of the mTOR signaling pathway in cardiomyocytes and neurons [34, 49]. The mTOR signaling pathway was activated after OGD/R or CIRI, but DDIT4 knockdown attenuates OGD/R-evoked ischemic injury in neurons by inhibiting mTOR-mediated excessive autophagy [34]. In this study, C2dat2 knockdown dramatically restrained the increase in OGD/R-induced DDIT4 and Beclin-1 levels and LC3B II/I ratio and the down-regulation of P62 and p-mTOR/mTOR and p-P70S6K/P70S6K ratio, indicating that C2dat2 down-regulation suppressed autophagy via the miR-30d-5p/DDIT4/mTOR axis in CIRI. Together, these data indicate that C2dat2 up-regulation may have an important role in facilitating autophagy and apoptosis in CIRI.

Recent evidence suggests that these small RNAs (siRNAs or miRNAs) need to form effector ribonucleoprotein complexes (RISC) to fulfill their functions by directly silencing their target mRNAs as guides for RISC [26]. The HSC70/HSP90 chaperone machinery serves as a dynamic driving force for the entire RISC assembly pathway [26, 27]. In this study, C2dat2 was confirmed to bind with HSC70 and HSP90. Thus, it was hypothesized that CIRI induces the up-regulated C2dat2 to bind with HSC70/HSP90, blocks the RISC assembly, reduces the “chaperone catalytic engine” to load miR-30d-5p duplexes into Ago proteins, and inhibits the miR-30d-5p from silencing their target mRNA DDIT4 and consequently inhibits the mTOR signaling pathway protein phosphorylation and facilitates neuronal autophagy and apoptosis. However, some potential limitations of this study should be discussed. Although it was confirmed that C2dat2 bound with the HSC70/HSP90 conjugate, the exact mechanisms involved in this remained unclear. Further studies should also focus on the interplay among C2dat2, the miR-30 family, and HSC70/HSP90 in brain CIRI. These observations advance the understanding of the mechanisms of CIRI and may enrich the ceRNA hypothesis in CIRI.

In summary, this study reveals that lncRNA C2dat2 facilitates autophagy and apoptosis via the miR-30d-5p/DDIT4/mTOR axis in CIRI, improving the understanding of the regulatory mechanism of C2dat2 in CIRI and indicating the potential therapeutic target for ischemic stroke treatment.

## MATERIALS AND METHODS

### Bioinformatics analysis

For the expression profile data, H1206140A provided by Kangcheng Shanghai (Shanghai, China) was used. The correlation among C2dat2, miRNA, and mRNA expression in ischemic stroke was examined by Spearman’s correlation test. Using these related records, a subnetwork of the lncRNA C2dat2 was then drawn using the Cytoscape version 2.8.3 tool (http://www.cytoscape.org/). All predicted target mRNAs were analyzed through the GO term and Kyoto Encyclopedia of Genes and Genomes (KEGG) pathway enrichment analyses (https://david.ncifcrf.gov/) [50–52]. *P* < 0.05 was considered statistically significant. Besides, a CNC network and a ceRNA network of C2dat1 and C2dat2 in CIRI were also constructed using the Cytoscape version 2.8.3 tool, respectively.

### Animals and mouse MCAO model

C57BL/6J mice (8–9 weeks of age, 22–25 g in weight) obtained from Vital River Laboratory Animal Co. (Beijing, China) were used for the MCAO model operation, as described previously [36]. Briefly, after anesthesia with isoflurane mixed with O2 and N2, a 6-0 nylon monofilament suture was gently inserted from the external carotid artery up to the internal carotid artery. After 1 h occlusion, the filament was slowly removed for reperfusion at 3, 6, 12, or 24 h, with the external carotid artery tied enduringly. After reperfusion, mice were deeply anesthetized, and the brains were obtained for further analysis. All animal experiments were conducted according to the protocol approved by the Nanyang Normal University for the Care and Use of Laboratory Animals. The mouse neurological function after CIRI was assessed using Clark scores [36].

### Cell culture and OGD/R injury

Mouse N2a cells were purchased from the American Type Culture Collection (Manassas, VA, USA) and cultured in high-glucose Dulbecco’s modified Eagle’s medium (Gibco, Waltham, MA, USA) comprising 10% fetal bovine serum in a humidified atmosphere of 5% CO2 at 37°C. To model OGD/R injury, N2a cells were placed into an anaerobic chamber (Forma Scientific) comprising a hypoxic gas mixture (5% CO2 + 95% N2) for 3 h [35]. Thereafter, cells were cultured in a high-glucose medium under normal conditions for reoxygenation.

### Sample preparation

The ischemic penumbra and ischemic cortex were taken as experimental samples, as described previously [26]. Briefly, the brain cerebral cortex was sectioned into three slices: section 1 was the ischemic cortex, section 2 was the ischemic penumbra, and section 3 was the rest of the brain tissue. Sections 1 and 2 were used for RNA or protein extraction. For all brain tissues and N2a cells, samples were snap frozen and stored at -80°C.

### RNA extraction and RT-qPCR

Total RNA extraction for N2a cell and mouse tissue was performed using the TRIzol LS reagent [36]. Then, the RNA integrity and purity of the samples were assessed. For miRNA analysis, RT-qPCR was performed using the Bulge-loop miRNA qRT-PCR Starter kit (RiboBio Co., Ltd., Guangzhou, China). miRNA quantification was determined using the Bulge-loop miRNA qRT-PCR Primer set (one RT primer and a pair of qPCR primers for each set) specific for miR-30d-5p, designed by RiboBio. U6 small nuclear RNA was used as an endogenous control for normalization. For lncRNA and mRNA analysis, total RNA (1 μg) was used to generate cDNA using the iScript cDNA synthesis kit (Forma Scientific, Marietta, OH, USA). Real-time PCR was then performed with SYBR Green Real-time PCR Master Mixes (Thermo Fisher Scientific, Waltham, MA, USA) on CFX96 Real-time PCR Detection System (Bio-Rad, Hercules, CA, USA) to analyze the expression of C2dat2. Data were normalized using glyceraldehyde 3-phosphate dehydrogenase (GAPDH) as control.

### Cell Survival assay

The CCK-8 assay was used to examine the survival of N2a cells after OGD/R. Briefly, N2a cells were plated at a low density (1 × 105 cells/well in 96-well plates). Then, the CCK-8 solution was added to the cells in each well followed by incubation after treatment. The survival rates of N2a cells after OGD/R relative to normoxia were calculated by measuring optical density at 450 nm [36].

### RACE

RACE was analyzed using the SMARTer RACE cDNA Amplification kit (Clontech, Mountain View, CA, USA) according to the manufacturer’s protocol to identify the transcriptional initiation and termination sites of lncRNA C2dat2.

### Nuclear and cytoplasmic cell fractions

The nuclear and cytoplasmic fractions were analyzed as described previously [36]. N2a cells were harvested and washed with phosphate-buffered saline (PBS) and then resuspended in hypotonic lysis buffer (HLB). The supernatant obtained after the addition of sodium acetate and ethanol was precipitated at -20°C and stored overnight to obtain the cytoplasmic fraction. Then, the pelleted nuclei were washed with HLB and resuspended in modified Wuarin-Schibler (MWS) buffer. Finally, the samples were spun after incubation to obtain the nucleoplasmic fraction. The pelleted chromatin was washed with MWS buffer. All supernatants were used for RNA extraction with TRIzol LS reagent.

### Western blotting

Western blotting was conducted according to standard methods [6]. DDIT4, LC3B, P62, Beclin-1, p-mTOR, mTOR, p-P70S6K, and P70S6K were detected in mouse tissue and N2a cells. Staining was visualized using the ECL detection reagents. The intensity of individual protein bands was measured by the ImageJ software. The protein level of β-tubulin was loaded as the normalized control.

### FISH

RNA-FISH was performed with specific Cy3-labeled probes to visualize the location of C2dat2 expressed in the cortex. For each tissue sample, 2 mm sections were prepared from each paraffin block. Thereafter, each section was deparaffinized, pretreated, and protease digested using the FISH kit (RiboBio). Then, the C2dat2 FISH probe (red) was hybridized at 40°C for 12 h in the dark (RiboBio). The mouse brain slides were rinsed and counterstained with 4′, 6-diamidino-2-phenylindole (DAPI). Finally, the sections were mounted, and a coverslip was placed with a neat fluorescent mounting medium. Images were captured using a Leica microscope (Leica, Wetzlar, Germany).

### Dual-luciferase reporter assay

MUT and WT C2dat2 and MUT and WT 3′-UTR fragments of DDIT4 comprising the putative miR-30d-5p binding sites were synthesized at RiboBio. The constructs were cloned into the pmiR-RB-REPORT vector (RiboBio) with pmirGLO downstream of the luciferase reporter gene to generate C2dat2-WT, C2dat2-MUT, DDIT4-WT, and DDIT-MUT luciferase reporter. N2a cells were transfected with the luciferase reporter plasmids, miR-30d-5p inhibitor, miR-30d-5p mimic, inhibitor-NC, and mimic-NC for 48 h. The Renilla luciferase reporter was used as an internal control.

### RNA pull-down assay and mass spectrometry

lncRNA C2dat2 and its antisense RNA were transcribed from the vector pBluescript II SK(+) and biotin-labeled with the Biotin RNA Labeling Mix (Roche, Indianapolis, IN, USA) *in vitro*. Then, T7/T3 RNA polymerase was used via KpnI or XbaI single-enzyme digestion to prepare recombinant plasmids and linearized plasmid templates. Lysis buffer was added to the tissue sample of MCAO mice to lyse the cells by homogenization, after which the total tissue protein was prepared. Protein (1 mg) from MCAO mouse tissue was then mixed with biotinylated RNA (10 pmol). C2dat2 and its antisense RNA probes were incubated vertically with 1 mg total protein in RNA binding buffer by adding streptavidin beads. The beads were then collected and washed, after which the binding proteins were separated and silver-stained, and the excised gel bands were identified via mass spectrometry.

### Immunostaining and binary image analysis

Mouse tissue sections were blocked at room temperature for 60 min with a blocking solution comprising anti-Beclin-1 or anti-DDIT4 after deparaffinization. Then, the samples were washed with Tris-buffered saline for 30 min followed by incubation with secondary antibodies. The nuclei were counterstained with DAPI [35].

### TUNEL staining and nissl staining

Cellular apoptosis was quantified via TUNEL staining following the protocol of the One Step TUNEL Apoptosis Assay Kit (Keygen, Nanjing, China). The treated N2a cells and MCAO brain slices were stained according to the manufacturer’s instructions [36]. The survival rates of the neurons in the mouse penumbra were evaluated via Nissl staining. Briefly, the brain slices were stained with Nissl staining solution at 37°C for 10 min. Similarly, TUNEL-positive cells and typical Nissl bodies were counted under a fluorescence microscope (Olympus, Tokyo, Japan).

### Statistical analysis

All statistical analyses were performed using GraphPad Prism 7 (GraphPad Software, La Jolla, CA, USA). Statistical differences between two groups of data via *t*-test among multiple groups were determined by one-way analysis of variance. In all cases, *P* < 0.05 was considered statistically significant.

### Ethical approval

All procedures performed in studies involving animals were in accordance with the ethical standards of the institution or practice at which the studies were conducted.

## Supplementary Material

Supplementary Figures

Supplementary Table 1
