# Supplementary material for: lncRNA C2dat2 facilitates autophagy and apoptosis via the miR-30d-5p/DDIT4/mTOR axis in cerebral ischemia-reperfusion injury
Source: Aging (Albany NY). 2021 Apr 4;13(8):11315–35. doi: 10.18632/aging.202824 (PMC8109078; doi:10.18632/aging.202824)
Supplement: Supplementary Figures [file aging-13-202824-s001.pdf]

SUPPLEMENTARY FIGURES

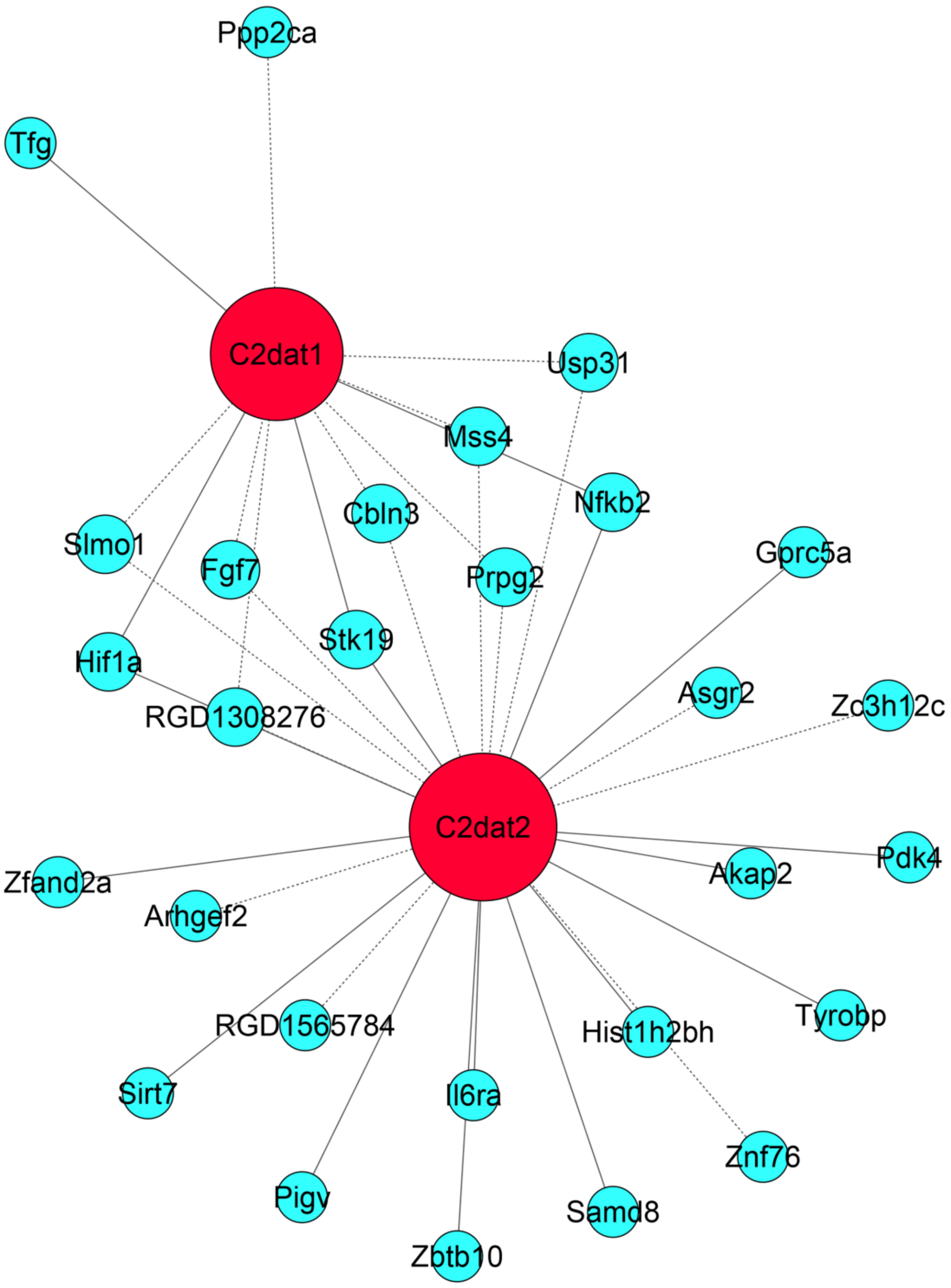

Supplementary Figure 1. CNC network between C2dat2 and C2dat1. Light-blue nodes are protein-coding RNAs, and red nodes are lncRNAs. The solid line represents a positive correlation, and the dashed line represents a negative correlation.

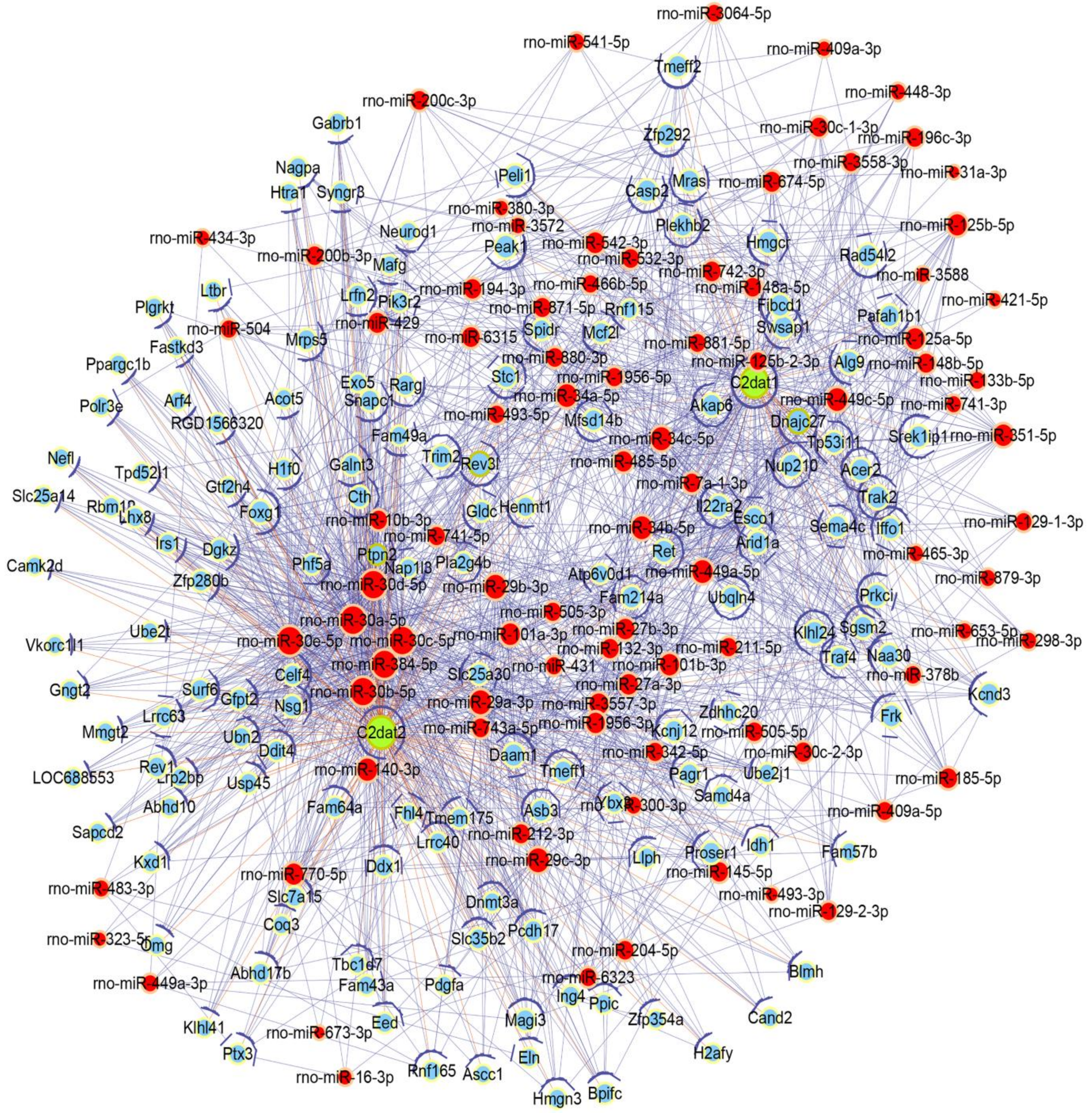

Supplementary Figure 2. ceRNA network between C2dat2 and C2dat1. Red nodes are miRNAs, light-blue nodes are protein-coding RNAs, and light-green nodes are noncoding RNAs. Edges with T-shaped arrow represent directed relationships, and edges without arrow represent undirected relationships.

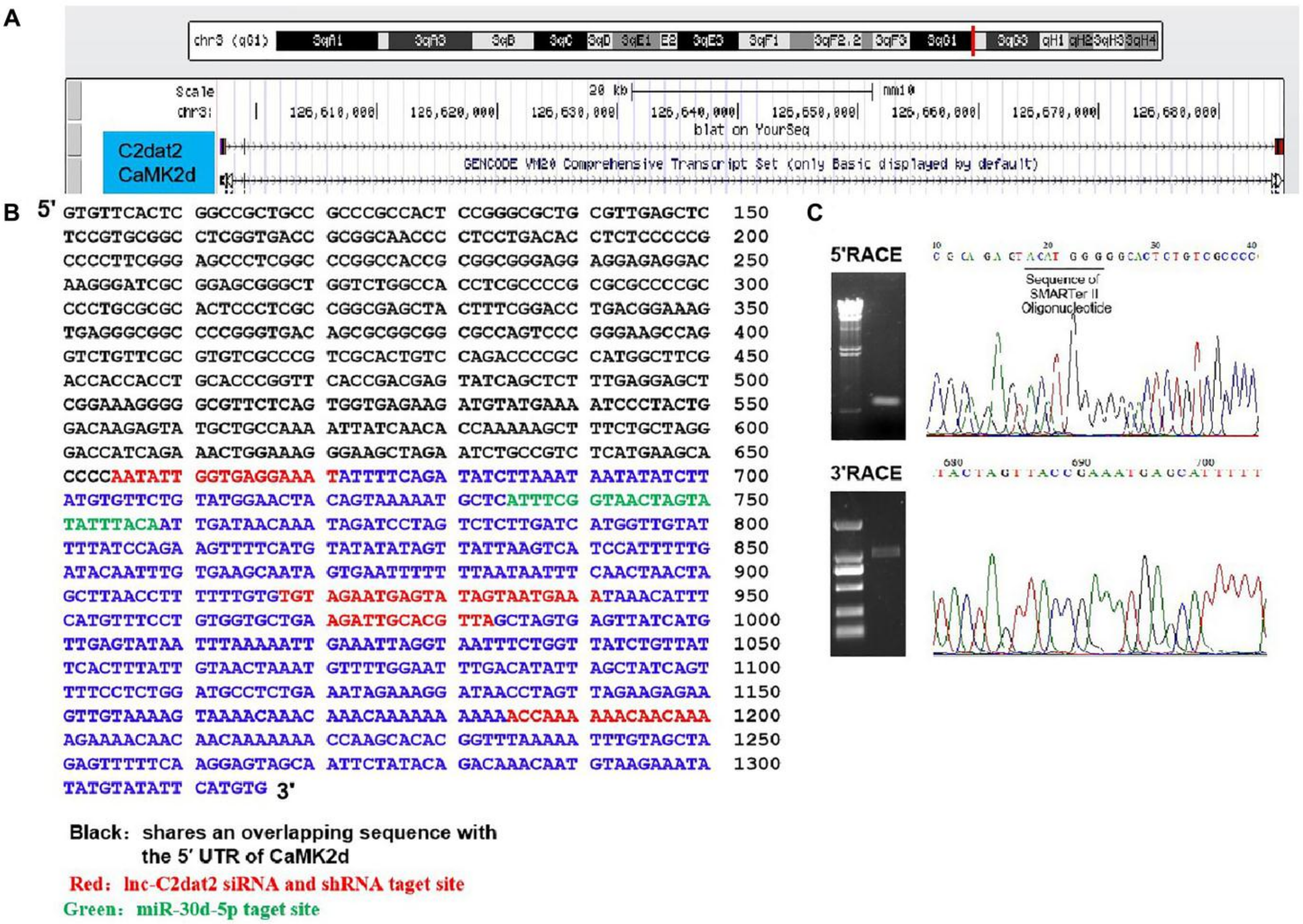

Supplementary Figure 3. Information about C2dat2. (**A** and **B**) C2dat2 is located on chromosome 3 (**A**) in the mouse genome and shares an overlapping sequence with the 5′-UTR of CAMK2d. siRNA, short hairpin RNA, and snoRNA were designed to target the region of C2dat2 outside of the overlapping sequence with the 5′-UTR of *CAMK2D*. (**B**) The full-length sequence of lncRNA C2dat2 was obtained by RACE. The nucleotide sequence of the full-length C2dat2 is given on the right. (**C**) Agarose gel electrophoresis and sequencing of the PCR product from the 5′-RACE and 3′-RACE procedure revealed putative transcriptional start and end sites of C2dat2.
